# Expression and *In Situ* Localization of Two Major PR Proteins of Grapevine Berries during Development and after UV-C Exposition

**DOI:** 10.1371/journal.pone.0043681

**Published:** 2012-08-24

**Authors:** Steven Colas, Damien Afoufa-Bastien, Lucile Jacquens, Christophe Clément, Fabienne Baillieul, Florence Mazeyrat-Gourbeyre, Laurence Monti-Dedieu

**Affiliations:** Laboratoire de Stress, Défenses et Reproduction des Plantes, Unité de Recherche Vignes et Vins de Champagne - EA 4707, UFR Sciences Exactes et Naturelles, Université de Reims Champagne-Ardenne, BP 1039, Reims, France; Consejo Superior de Investigaciones Cientificas, Spain

## Abstract

In grapevine *Vitis vinifera* L. cv Pinot noir, the Pathogenesis-Related (PR) proteins CHI4D and TL3 are among the most abundant extractable PR proteins of ripe berries and accumulate during berry ripening from véraison until full maturation. Evidence was supplied in favor of the involvement of these two protein families in plant defense mechanisms and plant development. In order to better understand CHI4D and TL3 function in grapevine, we analyzed their temporal and spatial pattern of expression during maturation and after an abiotic stress (UV-C) by *in situ* hybridization (ISH) and immunohistolocalization. In ripening berries, *CHI4D* and *TL3* genes were mainly expressed in the exocarp and around vascular bundles of the mesocarp. In UV-C exposed berries, *CHI4D* and *TL3* gene expression was strongly induced before véraison. Corresponding proteins localized in the exocarp and, to a lesser extent, around vascular bundles of the mesocarp. The spatial and temporal accumulation of the two PR proteins during berry maturation and after an abiotic stress is discussed in relation to their putative roles in plant defense.

## Introduction

Plants respond to environmental stimuli and pathogen attacks by inducing a variety of defense mechanisms. Among these, defense proteins, termed as pathogenesis-related (PR) proteins, are highly accumulated in all plant tissues and organs. PR proteins were first described in tobacco leaves, following infection with tobacco mosaic virus [1], [2] and have subsequently been identified in numerous monocotyledonous and dicotyledonous plants, and hence can be considered as ubiquitously distributed in plant kingdom [3]. In grapevine (*Vitis vinifera* L.) different families of PR proteins have been found to be induced in different organs after a stress or at specific developmental stages [4], [5], [6], [7], [8]. In berries, expression of genes encoding several PR proteins dramatically increases at the onset of berry softening (véraison) [5], [9]. These proteins are abundant in ripe berry and represent about 20% of the total proteins [10], [11]. Among these, chitinase (PR-3) and thaumatin-like (PR-5) proteins are the two major PR protein families represented in berry juice [12], [13], [14], [15]. Due to their resistance to proteolysis and stability at acidic pH, these proteins cause haze in wine and thus decrease its commercial value [16], [17]. In berries, the most abundant proteins belonging to these two PR protein families are one chitinase of class IV CHI4D/CHV5 (NCBI reference sequence: XP_002275386.1) and two thaumatin-like proteins, VvTL1 (NCBI reference sequence: XP_002282910.1) and VvTL2/TL3 (NCBI reference sequence: XP_002282964.1) [5], [18], [19]. However their concentration could vary depending on the cultivar and environmental conditions [14], [15], [20], [21]. This accumulation throughout maturation concomitant with the accumulation of sugars that are source of carbon for fungi could constitute a preformed defense. Indeed some chitinase and thaumatin-like proteins are known to have antifungal properties [18], [22], [23]. Chitinases known for degrading chitin, an insoluble homopolymer of *β*-(1–4)-linked N-acetylglucosamine units, may also perform signaling functions as releasing elicitors from invading fungal hyphae [24], [25]. Thaumatin-like proteins enhance the permeability of fungal cell membrane by forming holes, which enables water influx and causes rupture of hyphal membrane [26], [27]. In addition, some thaumatin-like proteins bind to β-1,3-glucans and display β-1,3-glucanase activity [28]. Furthermore, some evidence exists for chitinase and thaumatin-like developmental regulation in specific tissues and at specific stages during plant development suggesting that both proteins could be involved in other functions than plant defense [29], [30], [31], [32]. For example, PR-5 overexpression was shown to improve *Arabidopsis* seed germination [33]. In order to better understand chitinase and thaumatin-like protein roles in grape berry maturation and/or defense, it is not only necessary to monitor their temporal pattern of expression throughout development but also to identify the tissues where their expression occurs, which has never been performed before. For that purpose, *in situ* hybridization and immunohistolocalization are to our knowledge, the best suitable approaches.

The present report focuses on the temporal and spatial expression of *CHI4D* and *TL3* in the whole berry tissues at different developmental stages and after UV-C irradiation. This treatment allowed to study the incidence of a stress on morphologically preserved tissues contrary to fungus-infected berries where a tissue degradation follows fungal infection. Global expression of *CHI4D* and *TL3* was followed by qRT-PCR and western blot analyses and was associated to *in situ* hybridization and immunohistolocalization approaches. The biological function of CHI4D and TL3 is discussed in relation to their tissular localization and variation in expression levels during berry maturation and after UV-C stress.

## Materials and Methods

### Plant Material

Berries of *Vitis vinifera* L. cv Pinot noir were collected in vineyard at the experimental station of the Comité Champagne, Plumecoq (France) in 2008, 2009 and 2010. Four stages of development were identified according to the BBCH scale [34]: groat-sized berries (BBCH75), bunch closure (BBCH77), onset of véraison (BBCH81) and mature berries (BBCH89). All necessary permits were obtained for the described field studies.

### UV-C Treatment

Detached berries were placed on wet Whatman in Petri dishes and irradiated using a UV-C lamp (254 nm, Vilber Lourmat, Model VL-6.C, output 710 µW cm^−2^, 12 cm distant) for 7 min under agitation. Controls consisted of non-irradiated but agitated berries (named control) and non-irradiated and non-agitated berries (named untreated berries). Petri dishes were then sealed with parafilm and placed in a 22°C growth chamber (16/8 h photoperiod) for 48 and 96 h.

### RNA Extraction and Real-time Quantitative PCR

RNA extraction and real-time quantitative PCR were performed as previously described in Petit *et al.* (2009) [35] with minor modifications. A 150 ng aliquot of total RNA was reverse-transcribed using Verso reverse transcriptase (AR-4113/A, Thermo scientific). Denaturation was carried out at 95°C for 10 s and detection system used was Chromo 4 (Bio-Rad). The results were expressed as mRNA copy number/1000 *EF1-α* mRNA. Expression profiles were statistically analyzed by Mann-Whitney U test (P<0.05). Specificity of primers (Table 1) was checked with Primer-BLAST analysis (NCBI) and by sequencing the different amplicons.

**Table 1:** Primer sequences used for qRT-PCR.

Protein	Gene (accession no.)	Primer sequences	Product (bp)
Class IV chitinase(XP_002275386.1)	*CHI4D* (XM_002275350)	5′-CTACAACTATGGCGCTGCTG-3′ 5′-CCAAAACCATAATGCGGTCT-3′	109
Thaumatin-like(XP_002282964.1)	*TL3*(VvTL2) (XM_002282928)	5′-CCTAACACCTTAGCCGAATTCGC-3′ 5′-GGCCATAGGCACATTAAATCCATC-3′	93
Elongation factor 1α(XP_002284924.1)	*Vvef1α* (XM_002284888)	5′-GAACTGGGTGCTTGATAGGC-3′ 5′-AACCAAAATATCCGGAGTAAAAGA-3′	150

### *In situ* Hybridization

Specific primers (Table 2) were designed to generate template DNA (including 3′UTR of target gene) with T7 promoter tailed (5′-GCGAAAT-TAATACGACTCACTATAGGGAGA-3′) for synthesis of RNA probes according to Colas *et al*. (2010) [36]. Templates DNA were amplified from sequences subcloned into pGEM®-T easy vector (Promega) and submitted to BLAST search (NCBI) to confirm their specificity. Sense and antisense probes were labeled with UTP-digoxigenin during the transcription step. Preparation of berry samples, hybridization and signal detection were performed as described in Colas *et al.* (2010) [36].

**Table 2:** Primers used to generate template DNA for synthesize of RNA probes.

Gene (accession no.)	Primer sequences	Product (bp)
*CHI4D* (XM_002275350)	5′-CTTGAGCAACCCTGGAATTGTT-3′5′-GAGCATTATATGGCAAAAGCCC-3	362
*TL3* (XM_002282928)	5′-GCTGCGCTAAAGACTACCGGT-3′ 5′-CTAGGCTTTTGGAACCCAAGG-3′	302

**Figure 1:**
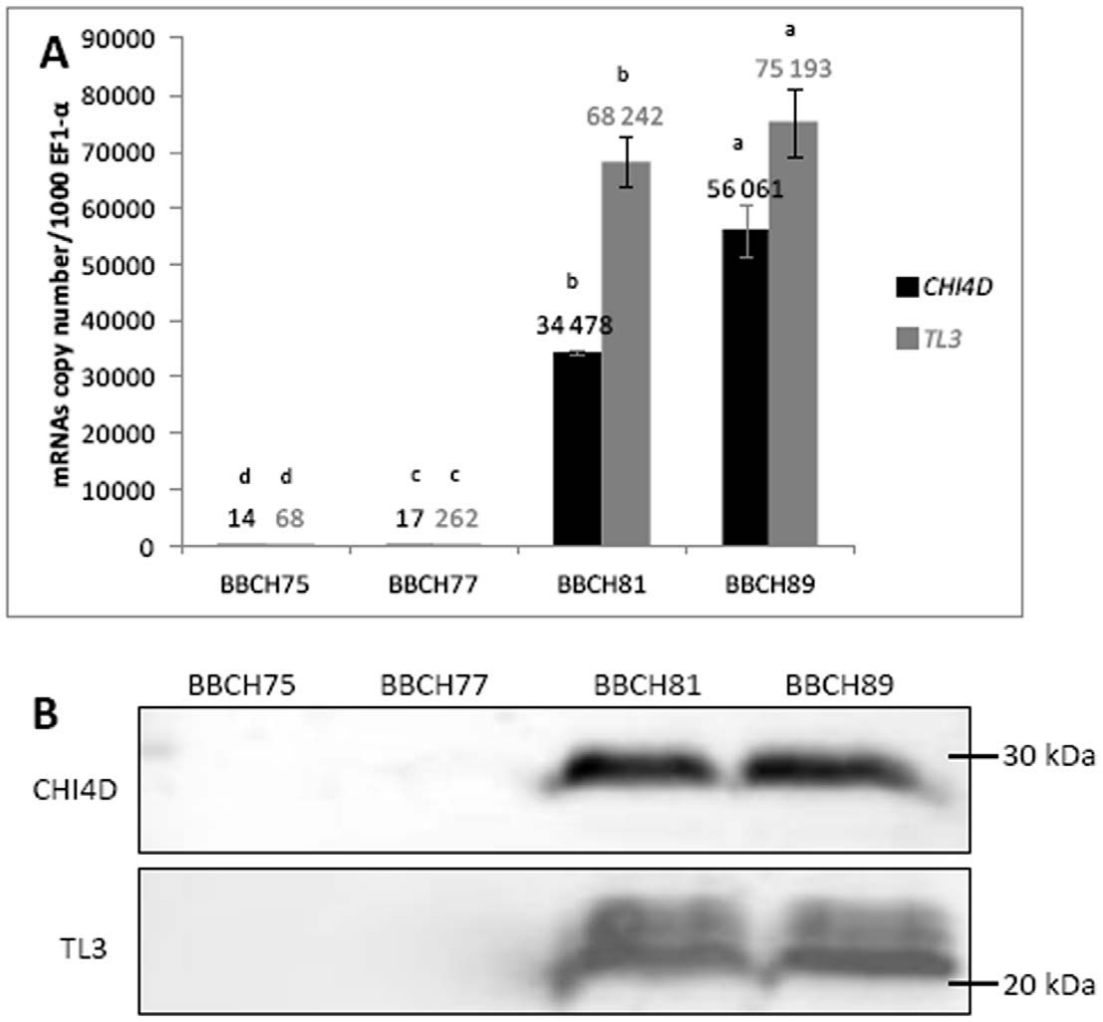
Global CHI4D and TL3 mRNAs/proteins expression in berry during development. **A)** Transcript accumulation of *CHI4D* and *TL3* genes in Pinot noir grapevine berries during development. Analyses were performed by quantitative RT-PCR. Levels of transcripts were calculated using the standard curve method from duplicate data, with grapevine *EF1-α* gene as internal control and were expressed as mRNA copy number/1000 *EF1-α* mRNAs. Values represent the mean ± SD of duplicates of the harvest 2009. Letters indicate significant differences between BBCH stages as calculated by Mann-Whitney U test (P<0,05). The same trend was observed for 2008 and 2010. **B)** Western blot analysis of CHI4D and TL3 in Pinot noir berries during maturation. Results represent the harvest of 2009 the same trend was observed for 2008 and 2010.

### Protein Extraction and Western Blotting

Berries were ground in liquid nitrogen to a fine powder. Extraction of proteins was realized according to Castro *et al.* (2005) [37] but proteins were precipitated for 1 night at −20°C. Total protein content was estimated for each sample according to Fey *et al*. (1997) [38] using BSA as standard. Two point five µg of proteins were diluted in Laemmli buffer [39] (2% SDS (w/v), 62.5 mM Tris-HCl pH 6.8, 10% glycerol (v/v), 1 M β-mercaptoethanol, 0.001% bromophenol blue (w/v)) and denatured at 95°C for 5 min. Proteins were separated by 15% SDS-PAGE electrophoresis at 20°C using the Mini-PROTEAN® Tetra Cell (Bio-Rad) at 200 V for 1 h and transferred on a polyvinylidene fluoride (PVDF) membrane for 7 min using I Blot gel transfer System (Invitrogen). The PVDF membrane was incubated for 1 h with TBST (20 mM Tris-HCl, 500 mM NaCl at pH 7.5, 0.05% Tween-20 (v/v)) containing 3% (w/v) of powdered milk to saturate the remaining protein binding sites. Membrane was incubated for 1 h with antibodies diluted in TBST with milk (1∶10000 for CHI4D and 1∶250 for TL3) and then with goat anti-rabbit IgG-horseradish peroxidase conjugate (1∶3000) (Bio-Rad). Immunoreacting bands were detected with ChemiDoc™ XRS by treatment with SuperSignal® west pico chemiluminescent substrate (Pierce) according to the manufacturer’s protocol. Polyclonal antibodies used in this study were raised against chitinase (CHI4D) and thaumatin-like (TL3) purified from Pinot noir grape berries by Manteau *et al*. (2003) [13].

**Figure 2:**
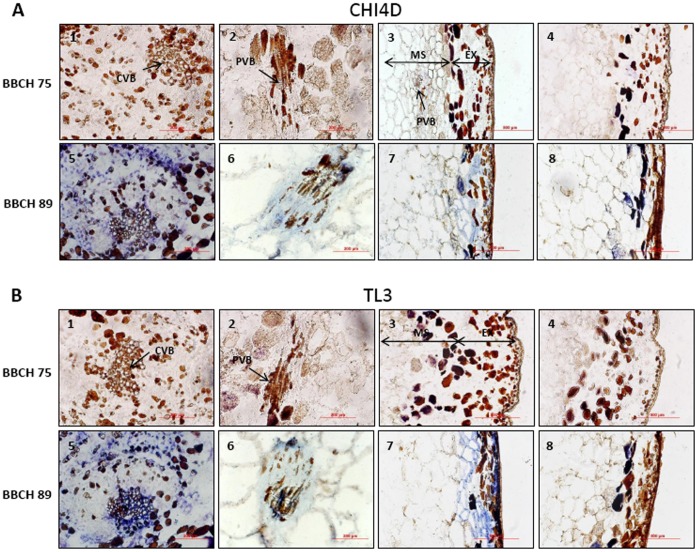
Localization of CHI4D and TL3 mRNAs expression sites in berry tissues during development. *In situ* localization of *CHI4D* **A)** and *TL3* **B)** mRNAs in Pinot noir berry tissues at BBCH75 (1, 2, 3, 4) and BBCH89 (5, 6, 7, 8) stages. Blue signal indicates the location of antisense (1, 2, 3, 5, 6, 7) and control sense (4, 8) probes. Sense probe controls indicate background staining. 1 and 5: central vascular bundles (CVB) in the mesocarp (MS), 2 and 6: peripheral vascular bundles (PVB) in the mesocarp, 3, 4, 7 and 8: exocarp (EX).

### Immunohistolocalization

Immunofluorescence analysis was carried out according to Colas *et al*. (2010) [36] on samples prepared in the same conditions as used for *in situ* hybridization concerning BBCH stage, treatment (UV-C or not) and tissue preparation. Sections were incubated with the primary anti-CHI4D (1/200) or anti-TL3 antibodies (1/200) [13]. Immunolabelling was detected after 1 h incubation at room temperature with the secondary antibodies Fluoprobes^©^ 488 (Interchim) at a dilution of 1/250. Control sections were incubated only with the secondary antibody. Images were recorded using an LSM 710 confocal laser microscope system (ZEISS, Jena, Germany) equipped with an Axio-observer Z1 inverted microscope and a Plan-apochromat 20x/0.8 DIC. The 488 nm excitation line from Argon ion laser (0.8% power) was used to excite fluorescence of Fluoprobes^©^ 488 and to record interference contrast images. The emitted fluorescence of Fluoprobes^©^ 488 was transmitted in spectral detector including 32 photomultiplicators through dichroic beam splitter MBS 488. First, emitted spectra of Fluoprobes^©^ 488 and specimen autofluorescence were recorded in this experimental configuration. Then, “spectral unmixing” mode of the confocal microscope is running to separate images respectively due to Fluoprobes^©^ 488 and autofluorescence by spectral deconvolution of recorded signal. For each sample, Z stacks (n = 20 to 50) were acquired with Z step of 1 µm.

**Figure 3:**
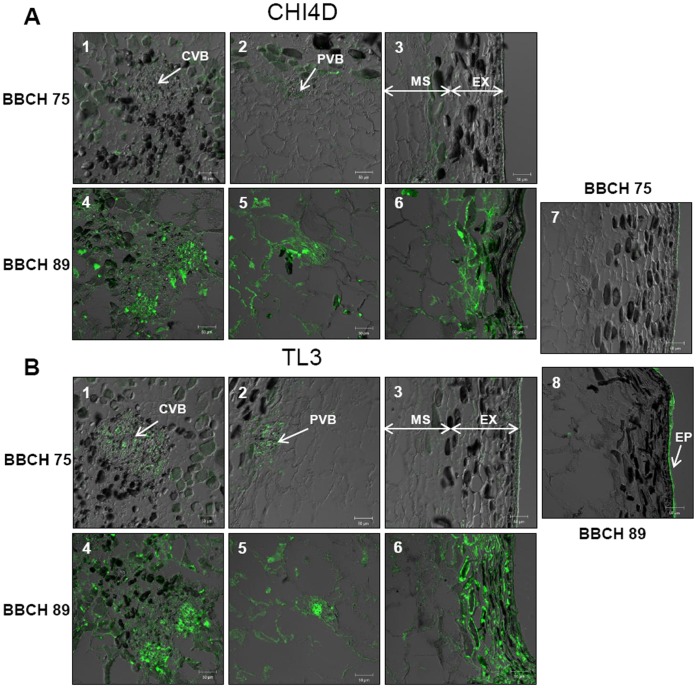
Localization of CHI4D and TL3 proteins expression sites in berry tissues during development. Immunolocalization of CHI4D **A)** and TL3 **B)** proteins in Pinot noir berry tissues at BBCH75 (1, 2, 3) and BBCH89 (4, 5, 6) stages. Controls without primary antibody indicate tissue background autofluorescence in berry tissues at BBCH75 (7) and BBCH89 (8) stages. 1 and 4: central vascular bundles (CVB) in the mesocarp (MS), 2 and 5: peripheral vascular bundles (PVB) in the mesocarp, 3, 6, 7 and 8: exocarp (EX). Epicuticular tissue (EP).

## Results

### Global *CHI4D* and *TL3* Expression in Grape Berry during Development

Expression of *CHI4D* and *TL3* genes was investigated in the whole berry by qRT-PCR at four developmental stages (Fig. 1A). Before véraison, at BBCH75 and BBCH77 stages, both genes were weakly expressed even though *TL3* was more expressed than *CHI4D*. During ripening stages (BBCH81 and BBCH89), expression greatly increased for both genes in comparison to the previous stages. This induction was approximately 4000- and 1100-fold respectively at full ripe, for *CHI4D* and *TL3* compared with the BBCH75 stage.

**Figure 4:**
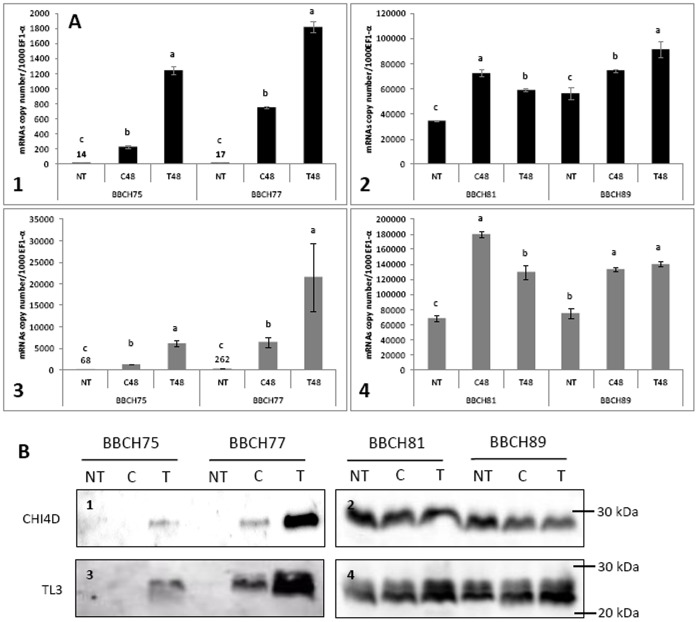
Global CHI4D and TL3 mRNAs/proteins expression in UV-C treated berries. A) Transcript accumulation of *CHI4D* (1, 2) and *TL3* (3, 4) genes in Pinot noir grapevine berries 48 h after UV-C stress. Analyses were performed by quantitative RT-PCR. Levels of transcripts were calculated using the standard curve method from duplicate data, with grapevine *EF1-α* gene as internal control and were expressed as mRNA copy number/1000 *EF1-α* mRNA. Values represent the mean ± SD of duplicates of the harvest of 2009. Letters indicate significant differences between treatments at each BBCH stage as calculated by Mann-Whitney U test (P<0,05). The same trend was observed for 2008 and 2010. Abbreviations: untreated berries (NT), control detached berries (C), treated berries (T). **B)** Western blot analysis of CHI4D (1, 2) and TL3 (3, 4) in Pinot noir berries 48 h after an UV-C stress. Results represent the harvest of 2009. The same trend was observed for 2008 and 2010. Abbreviations: untreated berries (NT), control detached berries (C), treated berries (T).

By western blotting analyses, CHI4D and TL3 proteins were not detected before véraison (BBCH75 and BBCH77) probably due to a weak concentration whereas they strongly accumulated during ripening (BBCH81 and BBCH89) (Fig. 1B). The size of CHI4D was about 29 kDa and for TL3, two bands were detected at about 22 and 24 kDa.

**Figure 5:**
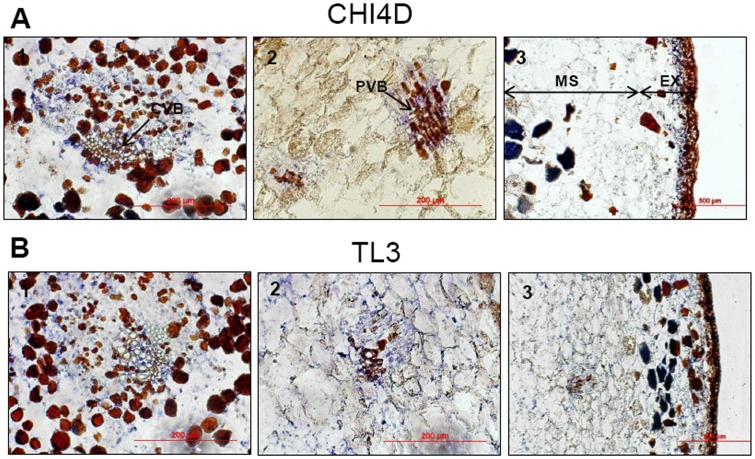
Localization of CHI4D and TL3 mRNAs expression sites in UV-C treated berries. *In situ* localization of *CHI4D* **A)** and *TL3* **B)** mRNAs in Pinot noir berry tissues at BBCH75 stages 48 h after UV-C irradiation. Blue signal indicates the location of antisense probes. 1: central vascular bundles (CVB) in the mesocarp (MS), 2: peripheral vascular bundles (PVB) in the mesocarp, 3: exocarp (EX).

### Localization of *CHI4D* and *TL3* Expression Sites in Berry Tissues during Development

The localization of the expression sites of *CHI4D* and *TL3* in berry tissues was carried out by *in situ* hybridization at the BBCH75 and BBCH89 stages because the global grape berry analysis showed maximal difference in expression levels at these stages for both genes. Control berry sections hybridized with *CHI4D* (Fig. 2 A: 4, 8) or *TL3* (Fig. 2 B: 4, 8) sense probes showed only little background staining on berry pigments. No signal was detected without probes (data not shown). At the BBCH75 stage, neither *CHI4D* nor *TL3* transcripts could be detected in the exocarp (Fig. 2 A: 3 and B: 3), the mesocarp (Fig. 2 A: 1, 2, 3 and B: 1, 2, 3) or in the pips (data not shown). At BBCH89 stage, *CHI4D* mRNAs were detected in the whole exocarp (Fig. 2 A: 7), in the mesocarp, mainly around the central vascular bundles (Fig. 2 A: 5) and in some cells associated with the peripheral vascular bundles (Fig. 2 A: 6). The same location was observed for *TL3* mRNAs, in the exocarp (Fig. 2 B: 7) and in the mesocarp, mainly around the central (Fig. 2 B: 5) and the peripheral vascular bundles (Fig. 2 B: 6). Concerning the pips, no *in situ* hybridization data could be obtained because of the difficulty to fix them in sections of ripe berries.

**Figure 6:**
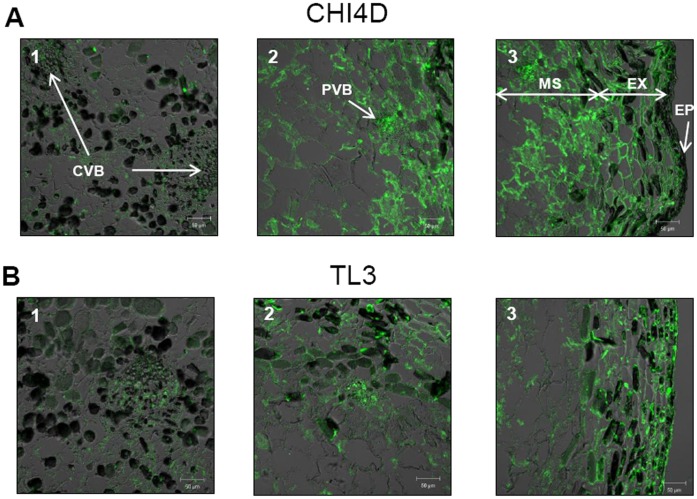
Localization of CHI4D and TL3 protein expression sites in UV-C treated berries. Immunolocalization of CHI4D **A)** and TL3 **B)** proteins in Pinot noir berry tissues at BBCH75 stages 48 h after an UV-C irradiation. Green fluorescence indicates the localization of proteins. 1: central vascular bundles (CVB) in the mesocarp (MS), 2: peripheral vascular bundles (PVB) in the mesocarp, 3: exocarp (EX). Epicuticular tissue (EP).

The localization of CHI4D and TL3 proteins was performed at the BBCH75 and BBCH89 stages using immunofluorescence. Controls without primary antibodies revealed faint backgrounds (Fig. 3∶7, 8) mainly associated with the epicuticular tissue. At the BBCH75 stage, CHI4D and TL3 were not detected in the exocarp (Fig. 3 A: 3 and B: 3) but were weakly detected in the mesocarp mainly around the central and the peripheral vascular bundles (Fig. 3 A: 1, 2 and B: 1, 2). In the pips, no signal was detected for CHI4D whereas a weak signal was observed for TL3 (data not shown). At the BBCH89 stage, CHI4D and TL3 strongly accumulated in the exocarp (Fig. 3 A: 6 and B: 6) and in the mesocarp, mainly associated with the central (Fig. 3 A: 4 and B: 4) and the peripheral (Fig. 3 A: 5 and B: 5) vascular bundles.

### Global *CHI4D* and *TL3* Expression in Grape Berry under UV-C Stress Condition

*CHI4D* and *TL3* gene expression was carried out by qRT-PCR analysis 48 h after an UV-C treatment at the four BBCH stages previously described (Fig. 4 A). Depending on the developmental stage, expression of *CHI4D* was induced to different levels in control detached berries as compared with untreated ones (from 1.3- to 44-fold) probably due to wounds caused by the berry detachment (Fig. 4 A: 1 and 2). In UV-C-treated berries, induction was higher compared to control and untreated berries (Fig. 4 A: 1) at BBCH75 (5.4- and 89-fold, respectively) and BBCH77 stages (2.4- and 107-fold respectively). However, at the BBCH81 and BBCH89 stages (Fig. 4 A: 2), very low induction in *CHI4D* gene expression level was observed in control detached (2.1- and 1.3-fold respectively) and UV-C treated berries (1.7- and 1.6-fold respectively) compared to untreated ones. Surprisingly, at BBCH81 stage induction was slightly stronger in control detached berries (1.2-fold) compared to UV-C treated ones probably due to heterogeneity of samples. Indeed, BBCH81 stage corresponds to the beginning of véraison with berries more or less colored. At this stage, berries of a same cluster might undergo asynchronous changes in numerous biological processes including alteration of defense responses to abiotic/biotic stimuli. As observed for *CHI4D*, expression of *TL3* in control berries was also induced compared to untreated ones depending on the developmental stage (from 1.8- to 24.5-fold) (Fig. 4 A: 3 and 4). In UV-C treated berries induction was higher compared to control and untreated berries (Fig. 4 A: 3) at BBCH75 (5- and 91-fold, respectively) and BBCH77 stages (3.4- and 82-fold, respectively). As observed for *CHI4D*, at the BBCH81 and BBCH89 stages (Fig. 4 A: 4), very low induction in *TL3* gene expression level was observed in control (2.6- and 1.8-fold respectively) and UV-C treated berries (1.3- and 1.8-fold respectively) compared to untreated ones and induction was slightly stronger in control (1.3-fold) compared to UV-C at BBCH81 stage.

CHI4D and TL3 protein contents were followed after UV-C treatment using western blot analyses along the four maturation stages studied before. In control berries, both proteins were detected from the BBCH77 stage (Fig. 4 B). At BBCH75 and BBCH77 stages, CHI4D (Fig. 4 B: 1) and TL3 (Fig. 4 B: 3) accumulated in treated berries but not in untreated ones. As previously described, two bands were visible for TL3 against a single one for CHI4D. Accumulation for both proteins was more intense at the BBCH77 than at the BBCH75 stage. CHI4D and TL3 accumulation was rather similar between BBCH81 and BBCH89 stages and between untreated, control and treated berries (Fig. 4 B: 2 and 4).

### Localization of *CHI4D* and *TL3* Expression Sites in Berry Tissues under UV-C Stress Condition

The localization of *CHI4D* and *TL3* mRNAs/proteins in berry tissues after UV-C exposure was investigated at the BBCH75 stage using *in situ* hybridization and immunofluorescence. Controls performed with *CHI4D* and *TL3* RNA sense probes showed only little background staining on berry pigments as shown in Fig. 2. Transcripts of *CHI4D* in UV-C treated berries were detected in the exocarp (Fig. 5 A: 3) and in association to the central (Fig. 5 A: 1) and peripheral vascular bundles (Fig. 5 A: 2) in the mesocarp. As for *CHI4D*, *TL3* mRNAs were detected in the exocarp (Fig. 5 B: 3) and around central and peripheral vascular bundles (Fig. 5 B: 1 and 2). However, unlike *CHI4D, TL3* mRNAs were detected in the whole mesocarp after an UV-C stress (Fig. 5 B: 3). No mRNA was detected in the pips neither for *CHI4D* nor for *TL3* (data not shown).

Immunofluorescence showed that CHI4D was widely spread in the exocarp (Fig. 6 A: 3) and in the mesocarp including sites around the peripheral vascular bundles (Fig. 6 A: 2) but weakly detected around the central vascular bundles (Fig. 6 A: 1) as in control (data not shown) and untreated berries (Fig. 3 A: 1). These observations concerning the mesocarp are however surprising compared to *in situ* hybridization experiments that showed a strong presence of *CHI4D* mRNAs mainly around the central (Fig. 5 A: 1) and peripheral (Fig. 5 A: 2) vascular bundles. TL3 proteins were detected in the exocarp (Fig. 6 B: 3) and in the mesocarp but they appeared less diffuse in this tissue than CHI4D (Fig. 6 B: 2) and accumulated more intensely around the peripheral vascular bundles. As observed with CHI4D, TL3 was barely detected around the central vascular bundles (Fig. 6 B: 1) as in control (data not shown) and untreated berries (Fig. 3 B: 1), while mRNAs had been obviously observed at these sites (Fig. 5 B: 1). At the BBCH89 stage no difference in tissular localization between untreated, control and UV-C-treated berries was observed for both proteins and their corresponding mRNAs (data not shown).

## Discussion

In this study, the spatial distribution of CHI4D and TL3, two major PR proteins in Pinot noir mature grape berries, and their corresponding transcripts, was investigated during berry maturation and under UV-C exposure. For these studies, if the specificity of the qPCR amplicons and ISH probes could be checked by sequencing (data not shown), the specificity of the antibodies used is still debatable. Chitinases and thaumatin-like proteins are encoded by multigenic families whose members organized within clusters share high sequence similarity in *Vitis vinifera* genome and it cannot be excluded that the polyclonal antibodies could recognize different isoforms although they were raised against purified proteins and that their specificity was successfully assessed as described by Manteau *et al*. (2003) [13]. Results based on qRT-PCR and western blot showed that *CHI4D* and *TL3* mRNAs/proteins accumulated from véraison to full maturation and that the regulation occurred at the transcriptional level. Similar patterns of expression were previously observed in Shiraz berry and Muscat of Alexandria [5], [21], [9]. In the present work we further showed that UV-C exposure induced *CHI4D* and *TL3* gene expression in Pinot noir berries before véraison. Previous studies also reported that some chitinase and thaumatin-like isoforms were induced by biotic or abiotic stresses [7], [35], [40]. In particular, *CHI4D* and *TL3* were induced by powdery mildew unlike *VvTL1* [7]. Concerning TL3, western analysis from Pinot noir berries revealed two bands with apparent masses of 22 and 24 kDa (Fig. 1 B and 4 B : 3, 4). The calculated molecular mass of TL3 without signal peptide is 21.2 kDa (http://web.expasy.org/compute_pi/), which corresponds to the lower band. As described above, we cannot exclude that the polyclonal antibodies raised against TL3 recognize another isoform but of higher molecular mass than TL3. However, it is possible that the higher band corresponds to a gycosylated form of the protein as described by Palmisano *et al*. (2010) [41] in white wine of *cv.* Chardonnay. In fact, according to *in silico* analysis of TL3 amino acids sequence this protein contains two potential N-glycosylation sites (NetNGlyc server). Furthermore, production of TL3 in *Pichia pastoris* resulted in the same two bands. In that case, PNGase treatment yielded a marked decrease of the larger one (unpublished results) suggesting that the putative glycosylation of the natural proteins cannot be ruled out. Therefore, we favour this second interpretation and assume that this TL3 protein is probably glycosylated *in vivo*.

Despite the abundant literature on chitinase and thaumatin-like protein expressions during ripening, only few studies have focused on the localization of their expression sites and such studies only related to dissected tissues: skin, pulp and pips. At the transcript level, in mature Shiraz berries, expression of *VvChi4/CHI4D* was localized in the pulp and skin [21] while in Cabernet Sauvignon mature berries, Grimplet *et al.* (2007) [42] using the Affymetrix GeneChip® approach, showed that *CHI4D* and *TL3* were expressed in the skin, but not in the pulp and that *TL3* mRNAs were also detected in the pips. The localization of chitinase and thaumatin-like expression sites could then vary according to the cultivar. Proteome analysis of Cabernet Sauvignon skin also revealed that chitinases accumulated in the exocarp during ripening [43]. Using a similar approach, Negri *et al.* (2008) [10] observed a sharp increase of chitinases and thaumatin-like proteins in the exocarp of Barbera cultivar throughout berry maturation.

Using *in situ* hybridization, we could for the first time in grapevine compare the expression sites of *CHI4D* and *TL3* genes in berry tissues during development and after UV-C stress. Both mRNAs were observed in the exocarp and in the mesocarp mainly around the vascular bundles of ripe berries. In other plants, chitinase and thaumatin-like transcripts were also observed around the vascular elements of various organs (stems, petals, roots) [30], [44]. Concerning the proteins, CHI4D and TL3 were detected at the same sites as their corresponding mRNAs but they were also observed in the whole mesocarp. The discrepancy between proteins and mRNAs in this tissue could be explained by sufficient amounts of proteins for immunofluorescence detection but insufficient amounts of transcript for *in situ* hybridization detection. Proteins could also spread *via* the apoplastic route and diffuse in the whole mesocarp [45]. *In silico* analysis of both proteins indicated that they contained a N-terminal signal peptide (http://bmbpcu36.leeds.ac.uk/prot_analysis/Signal.html) targeting mature proteins into the secretory pathway (http://www.cbs.dtu.dk/services/TargetP/) which supports this hypothesis. Moreover, in a previous study, chitinase exuded from cowpea roots (*Vigna unguiculata*) were also localized in the xylem cell wall vessel elements suggesting that the apoplast was a potential pathway to transport these proteins through the root [45].

In pre-véraison stressed berry (UV-C), *CHI4D* and *TL3* mRNAs were detected in the exocarp, around the central and peripheral vascular bundles of the mesocarp, and in the whole mesocarp for *TL3*. Both proteins localized in the exocarp and in the mesocarp including sites around the peripheral vascular bundles. They were not detected around the central vascular bundles 48 h after UV-C treatment, whereas an induction of both transcripts was observed. The delay of 48 h between stress and sampling analysis may be not sufficient for the production of both proteins. Hence, proteins began to be detected only about 96 h after stress (data not shown). Our results then suggest that both CHI4D and TL3 first accumulate in the exocarp and around the peripheral vascular bundles corresponding to sub-epidermal tissues directly exposed to the radiation. Proteins would accumulate later around the central vascular bundles of berry.

The localization of both proteins in the exocarp and around vascular elements could be strategic to inhibit/limit the penetration and/or the development of pathogenic agents. It is well known that some fungi like *Botrytis cinerea* could penetrate berries by the exocarp [46], [47] and that some bacteria like *Xylella fastidious* could propagate by the vessels of plants [48], [49]. Moreover, PR proteins secreted *via* the apoplastic route near vascular elements could be exported in the sap [50], [51], [52], [53]. The same temporal and spatial patterns of expression for *CHI4D* and *TL3* suggest that the corresponding proteins have complementary roles in berry. Chitinase and thaumatin-like proteins have been shown to play an important role in the plant defense mechanisms principally against fungi [29], [32]. *In vitro* inhibition of mycelial growth and conidia germination of *B. cinerea* were obtained with chitinase and thaumatin-like proteins extracted from berries of Pinot noir, Moscatel and Semillon [18], [22], [54]. Moreover berries at pre-véraison stage are highly susceptible to powdery mildew infection caused by *Erysiphe necator*, but become resistant to this pathogen during ripening [55], [56], as levels of CHI4D and TL3 increase. Transgenic grapevines expressing a rice chitinase exhibit resistance against *E. necator* and *Elisinoe ampelina*, the causal agent of anthracnose [57]. More recently, transgenic grapevines overexpressing the thaumatin-like VvTL1 protein showed less severe symptoms to *E. necator* on the leaves and enhanced rot resistance of ripe berries [58].

Several studies showed that chitinases and thaumatin-like are accumulated during ripening in a wide variety of fruits like cherimoya, pineapple, cherry, banana, Japanese pear or pepper [59], [60], [61], [62], [63], [64]. They could be involved in plant growth processes [29] and also in fruit maturation [65], [66]. As suggested by Edreva (2005) [3] these findings raise the question as to whether PR genes have evolved primarily to limit damage by invading pathogens, or have adapted from other function to serve an accessory protective role.

Owing to their temporal and spatial pattern of expression, chitinase and thaumatin-like could be key enzymes in berry defense mechanisms. Transgenic plants impaired in chitinase and thaumatin-like accumulation or accumulating high basal level of both proteins whatever the developmental stage of berry considered, could help to further characterize their role.
